# Supplementary material for: Effect of experimental hookworm infection on insulin resistance in people at risk of type 2 diabetes
Source: Nat Commun. 2023 Jul 26;14:4503. doi: 10.1038/s41467-023-40263-4 (PMC10372076; doi:10.1038/s41467-023-40263-4)
Supplement: Supplementary file 1 — Supplementary Information [file 41467_2023_40263_MOESM1_ESM.pdf]

Effect of experimental hookworm infection on insulin resistance in people at risk of Type 2 Diabetes: Supplementary Information

SUPPLEMENTARY TABLES

Supplementary Table 1. HOMA-IR change effect sizes at each evaluation visit

	Kruskal Wallis effect size	L3-20 vs Placebo	L3-40 vs Placebo
**6 months**	$\eta^2 = 0.04$<br>$d_{Cohen} = 0.42^S$	$r = 0.372$<br>$d_{Cohen} = 0.80^L$	$r = 0.235$<br>$d_{Cohen} = 0.48^S$
**12 months**	$\eta^2 = 0.16$<br>$d_{Cohen} = 0.86^L$	$r = 0.556$<br>$d_{Cohen} = 1.34^L$	$r = 0.341$<br>$d_{Cohen} = 0.73^M$
**18 months**	$\eta^2 = 0.23$<br>$d_{Cohen} = 1.08^L$	$r = 0.513$<br>$d_{Cohen} = 1.19^L$	$r = 0.088$<br>$d_{Cohen} = 0.18$
**24 months**	$\eta^2 = 0.01$<br>$d_{Cohen} = 0.20^S$	$r = 0.409$<br>$d_{Cohen} = 0.90^L$	$r = 0.195$<br>$d_{Cohen} = 0.40^S$

S, small effect size; M, medium effect size; L, large effect size.

Supplementary Table 2. Absolute values and changes in glycated haemoglobin (HbA1c/IFCC) in each treatment group

HbA1C IFCC (mmol/mol), Median and IQR (n)									
	**baseline**	**6 months**	**Δ6 mo.**	**12 months**	**Δ12 mo.**	**18 months**	**Δ18 mo.**	**24 months**	**Δ24 mo.**
**Placebo**	32<br>30-33 (*n = 12)*	34<br>30-36 (*n = 12)*	+1<br>0 to 3	34<br>30-37 (*n = 9)*	+2<br>0 to 4	33<br>30-36 (*n = 8)*	+3<br>0 to 4	35<br>33-37 (*n = 6)*	+4<br>1 to 5
**L3-20**	33<br>31-35 (*n = 13)*	32<br>30-34 (*n = 11)*	0<br>-1 to 1	32<br>31-35 (*n = 11)*	0<br>-1 to 1	33<br>32-36 (*n = 8)*	+1<br>0 to 2	33<br>32-37 (*n = 7)*	+2<br>1 to 4
**L3-40**	32<br>29-34 (*n = 11)*	31<br>30-35 (*n = 11)*	+1<br>0 to 3	33<br>32-35 (*n = 10)*	+3<br>1 to 4	**34 #**<br>33-37 (*n = 8)*	+4<br>2 to 7	32<br>31-37 (*n = 9)*	+2<br>2 to 5

Bolded data indicate presence of significantly different values, # Significantly different to baseline.

Supplementary Table 3. Absolute values and changes in serum cholesterol, high-density lipoprotein (HDL) and cholesterol/HDL ratio in each treatment group

	baseline	6 months	Δ6 mo.	12 months	Δ12 mo.	18 months	Δ18 mo.	24 months	Δ24 mo.
**Total cholesterol (mmol/L), Median and IQR (n)**									
**Placebo**	5.4<br>4.9-6.3 (*n = 12)*	4.9<br>4.4-6.0 (*n = 12)*	-0.4<br>-0.7 to 0.0	4.8<br>4.4.-5.4 (*n = 9)*	-0.4<br>-0.6 to -0.1	4.9<br>4.3-5.8 (*n = 8)*	-0.6<br>-1.1 to -0.1	5.0<br>4.5-5.3 (*n = 6)*	-0.2<br>-0.6 to 0.1
**L3-20**	5.2<br>4.6-6.3 (*n = 13)*	5.1<br>3.9-5.9 (*n = 12)*	-0.3<br>-0.6 to -0.2	5.0<br>4.2-5.6 (*n = 11)*	-0.2<br>-0.7 to 0.5	5.1<br>4.5-5.7 (*n = 10)*	-0.2<br>-0.4 to 0.4	4.9<br>3.9-6.8 (*n = 18)*	-0.2<br>-0.5 to 0.6
**L3-40**	5.4<br>4.9-5.5 (*n = 11)*	6.0<br>4.9-6.3 (*n = 11)*	+0.7<br>-0.2 to 0.9	5.6<br>4.8-5.7 (*n = 10)*	+0.2<br>-0.2 to 0.3	5.7<br>4.6-6.1 (*n = 8)*	**+0.4 ***<br>-0.2 to 0.6	5.3<br>4.3-5.8 (*n = 9)*	-0.1<br>-0.8 to 0.6
**HDL (mmol/L), Median and IQR (n)**									
**Placebo**	1.2<br>1.1-1.4 (*n = 12)*	1.1<br>1.0-1.3 (*n = 12)*	-0.1<br>-0.1 to 0.0	1.2<br>1.1-1.3 (*n = 9)*	0.0<br>0.0 to 0.1	1.2<br>1.1-1.6 (*n = 8)*	-0.1<br>-0.2 to 0.2	1.2<br>1.0-1.7 (*n = 6)*	0.0<br>-0.3 to 0.2
**L3-20**	1.2<br>1.0-1.5 (*n = 13)*	1.3<br>1.0-1.4 (*n = 12)*	-0.1<br>-0.2 to -0.1	1.1<br>1.0-1.4 (*n = 11)*	0.0<br>-0.1 to 0.0	1.2<br>1.0-1.3 (*n = 10)*	-0.2<br>-0.2 to 0.0	1.1<br>1.0-1.7 (*n = 8)*	+0.1<br>-0.1 to 0.2
**L3-40**	1.3<br>1.1-1.5 (*n = 11)*	1.3<br>1.1-1.5 (*n = 11)*	0.0<br>-0.2 to 0.2	1.3<br>1.1-1.3 (*n = 10)*	0.0<br>-0.1 to 0.1	1.2<br>1.1-1.3 (*n = 8)*	-0.1<br>-0.2 to -0.1	1.1<br>1.0-1.4 (*n = 9)*	-0.1<br>-0.3 to 0.0
**Total cholesterol/HDL ratio, Median and IQR (n)**									
**Placebo**	4.4<br>3.8-5.2 (*n = 12)*	4.0<br>3.8-4.9 (*n = 12)*	-0.1<br>-0.3 to 0.3	4.0<br>3.5 to 4.3 (*n = 9)*	-0.4<br>-0.6 to -0.2	3.9<br>3.3-4.6 (*n = 8)*	-0.2<br>-0.7 to 0.3	3.9<br>3.3-5.1 (*n = 6)*	+0.1<br>-0.6 to 0.6
**L3-20**	4.1<br>3.8-4.9 (*n = 13)*	4.2<br>3.7-4.9 (*n = 12)*	+0.2<br>0.0 to 0.3	4.2<br>3.7-5.3 (*n = 11)*	-0.1<br>-0.2 to 0.5	4.3<br>3.6-5.6 (*n = 10)*	+0.3<br>-0.3 to 1.1	4.2<br>3.3-5.0 (*n = 8)*	-0.2<br>-0.6 to 0.4
**L3-40**	4.0<br>3.7-4.9 (*n = 11)*	4.4<br>4.0-4.7 (*n = 11)*	+0.5<br>-0.2 to 0.8	4.5<br>3.9-5.1 (*n = 10)*	+0.1<br>-0.4 to 0.8	4.8<br>4.1-5.0 (*n = 8)*	+0.5<br>0.0 to 0.9	4.3<br>4.0-5.4 (*n = 9)*	+0.2<br>-0.1 to 0.7

Bolded data indicate presence of significantly different values, # Significantly different to baseline. *Significantly different to Placebo.

Supplementary Table 4. Absolute values and changes in serum Triglyceride and Low-Density Lipoprotein (LDL) levels in each treatment group

	baseline	6 months	Δ6 mo.	12 months	Δ12 mo.	18 months	Δ18 mo.	24 months	Δ24 mo.
**Triglycerides (mmol/L), Median and IQR (n)**									
**Placebo**	1.4<br>0.9-2.0 (*n = 12)*	1.4<br>1.0-2.0 (*n = 12)*	+0.2<br>-0.4 to 0.6	1.3<br>0.9-1.6 (*n = 9)*	0.0<br>-0.2 to 0.3	1.4<br>1.0-1.9 (*n = 8)*	+0.3<br>-0.2 to 0.5	1.8<br>1.4-2.9 (*n = 6)*	+0.7<br>0.4 to 1.4
**L3-20**	1.3<br>0.9-1.8 (*n = 13)*	1.3<br>0.8-1.7 (*n = 12)*	-0.2<br>-0.3 to 0.2	1.2<br>1.0-1.5 (*n = 11)*	-0.1<br>-0.4 to 0.2	1.4<br>0.8-2.5 (*n = 10)*	0.0<br>-0.2 to 0.8	1.1<br>0.9-1.9 (*n = 8)*	+0.1<br>-0.3 to 0.3
**L3-40**	1.4<br>0.8-2.0 (*n = 11)*	1.3<br>1.0-2.7 (*n = 11)*	-0.2<br>-0.4 to 0.9	1.8<br>1.0-2.6 (*n = 10)*	+0.1<br>-0.4 to 0.6	1.7<br>1.1-1.9 (*n = 8)*	-0.1<br>-0.2 to 0.7	1.3<br>0.9-2.1 (*n =9)*	+0.1<br>-03 to 0.3
**LDL (mmol/L), Median and IQR (n)**									
**Placebo**	3.5<br>3.0-4.4 (*n = 12)*	3.1<br>2.6-3.9 (*n = 12)*	-0.2<br>-0.7 to 0.0	**2.9 #**<br>2.6-3.4 (*n = 9)*	-0.3<br>-0.8 to -0.1	**2.9#**<br>2.5-3.5 (*n = 8)*	-0.6<br>-0.8 to -0.3	2.7<br>2.6-3.2 (*n = 6)*	-0.6<br>-0.7 to 0.2
**L3-20**	3.3<br>2.9-4.3 (*n = 13)*	3.4<br>2.5-3.8 (*n = 12)*	-0.4<br>-0.6 to 0.0	3.2<br>2.7-3.7 (*n = 11)*	-0.1<br>-0.6 to 0.6	3.1<br>2.6-3.9 (*n =10)*	-0.3<br>-0.5 to 0.5	3.1<br>2.1-4.1 (*n = 8)*	-0.2<br>-0.4 to 0.3
**L3-40**	3.4<br>2.6-3.6 (*n = 11)*	3.4<br>3.0-4.0 (*n = 11)*	+0.3<br>-0.3 to 1.3	3.2<br>3.0-3.8 (*n = 10)*	+0.2<br>-0.3 to 0.4	3.8<br>2.9-4.0 (*n = 8)*	**+0.5***<br>0.0 to 0.6	3.1<br>2.7-3.7 (*n = 9)*	+0.1<br>-0.5 to 0.5

Bolded data indicate presence of significantly different values, # Significantly different to baseline. *Significantly different to Placebo

SUPPLEMENTARY FIGURES AND LEGENDS

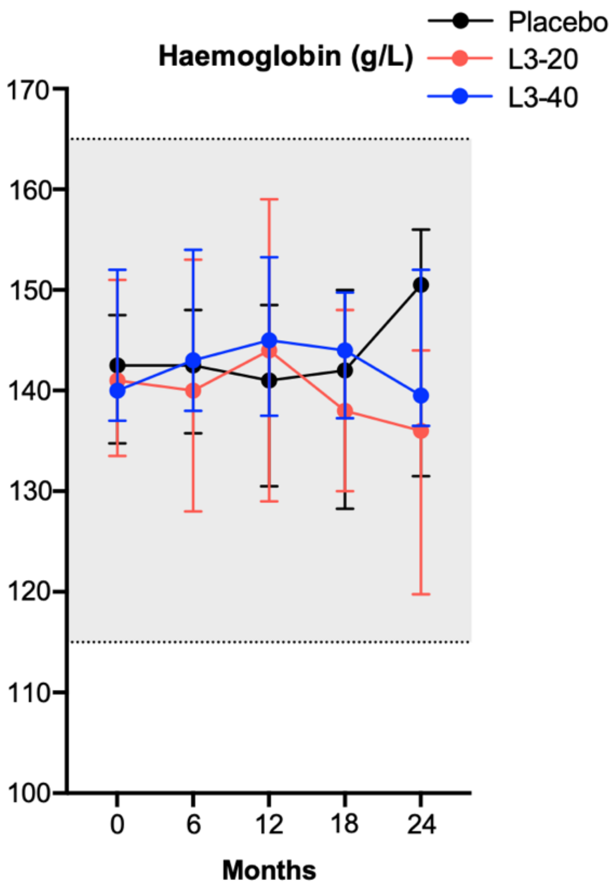

Supplementary Figure 1. Hookworm infection was not associated with anaemia. Blood hemoglobin concentration at each evaluation visit (median and IQR), shaded area indicates combined normal range for males and females. (Sample size for month 0, 6, 12, 18 and 24: Placebo n=12, 12, 9, 8 and 6; L3-20 n=13, 12, 11, 11 and 8; L3-40 n=11, 11, 10, 8 and 8, respectively). Source data are provided as a Source Data File.

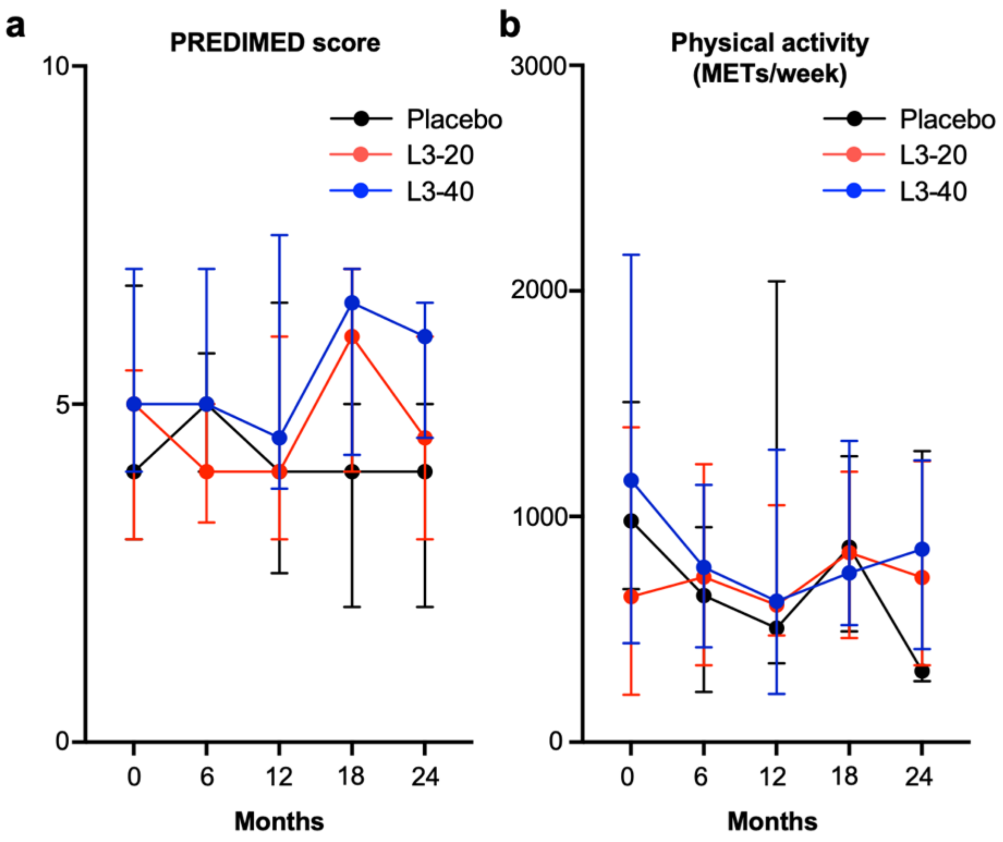

Supplementary Figure 2. Diet (PREDIMED score) and Physical Activity remained relatively stable in all groups throughout the study. (A) PREDIMED total scores in each treatment group at each evaluation visit (median and IQR). (Sample size for month 0, 6, 12, 18 and 24: Placebo n=12, 12, 9, 8 and 7; L3-20 n=13, 12, 12, 10 and 6; L3-40 n=11, 11, 10, 8 and 9, respectively). (**B**) Physical activity (Metabolic Equivalents of task, METs) per week assessed at each evaluation visit (median and IQR). (Sample size for month 0, 6, 12, 18 and 24: Placebo n=12, 12, 9, 8 and 7; L3-20 n=13, 12, 11, 10 and 8; L3-40 n=11, 11, 10, 8 and 9, respectively). Source data are provided as a Source Data File.

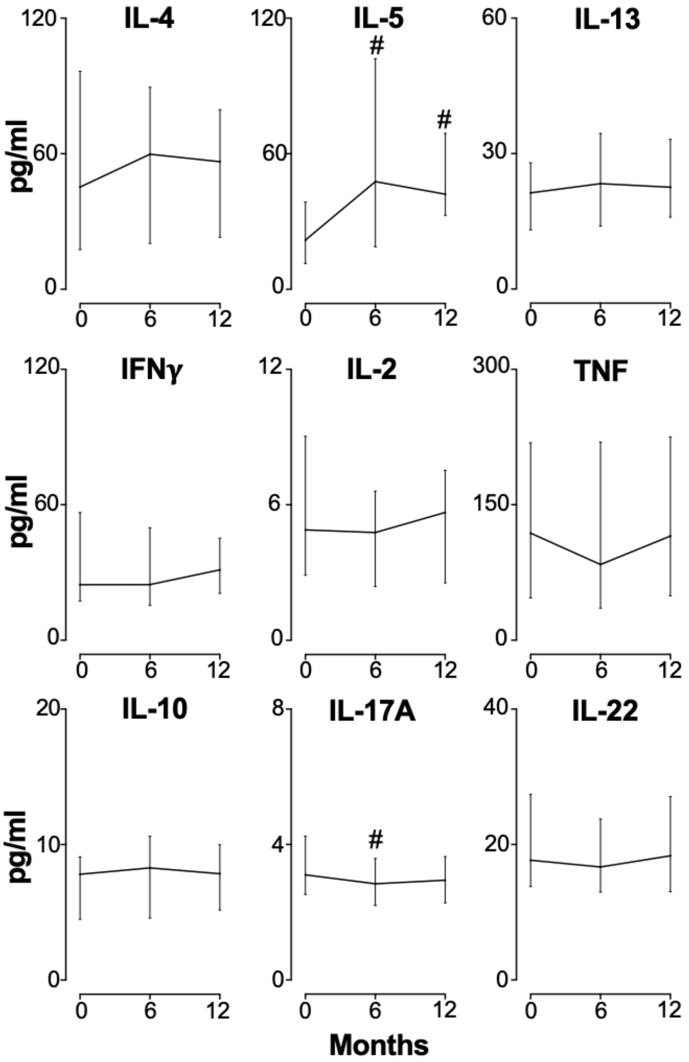

Supplementary Figure 3. Serum cytokine levels in combined hookworm-treated cohorts. Serum samples taken from L3-20 and L3-40 participants (n=17) at baseline, 6 months and 12 months were thawed and batch analysed using a multiplex human cytokine panel. Levels of nine different cytokines are depicted as median and IQR. **#** Significant difference to month 0 baseline value, Friedman's test with Dunn's multiple comparisons. IL-5 p= 0.0001 (6 months) and p= 0.02 (12 months); IL-17A p=0.026 (6 months). Source data are provided as a Source Data File.

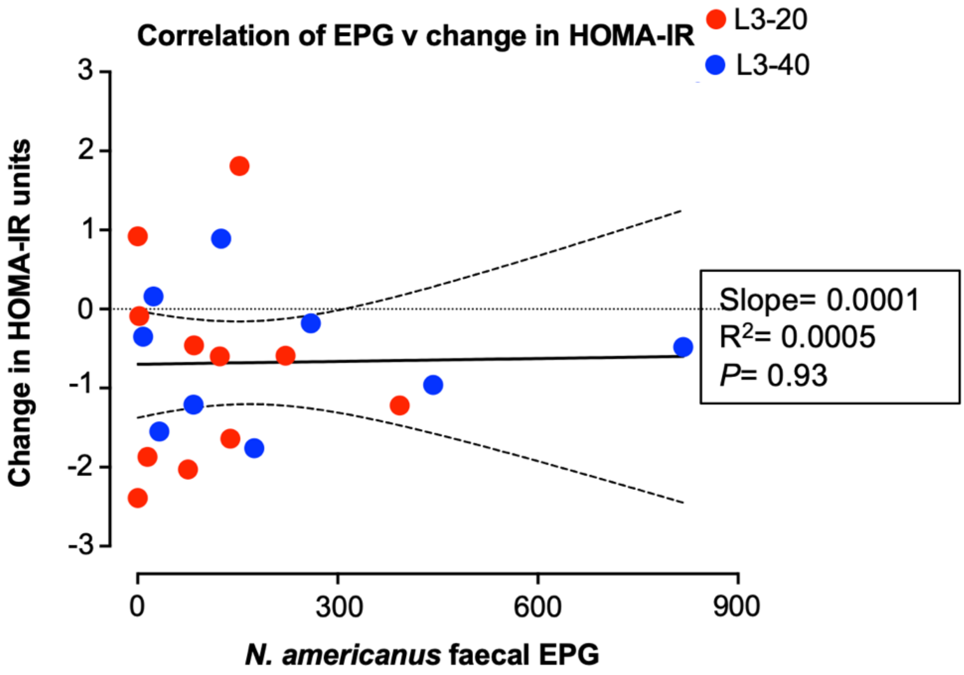

Supplementary Figure 4. Correlation analysis of hookworm infection intensity and changes in HOMA-IR. Hookworm eggs per gram (EPG) in faecal samples at 6 months were plotted against change in HOMA-IR from baseline to 6 months for L3-20 and L3-40 hookworm-treated participants. Simple linear regression analysis (solid line, dotted lines represent 95% CI) revealed no significant associations. Source data are provided as a Source Data File.

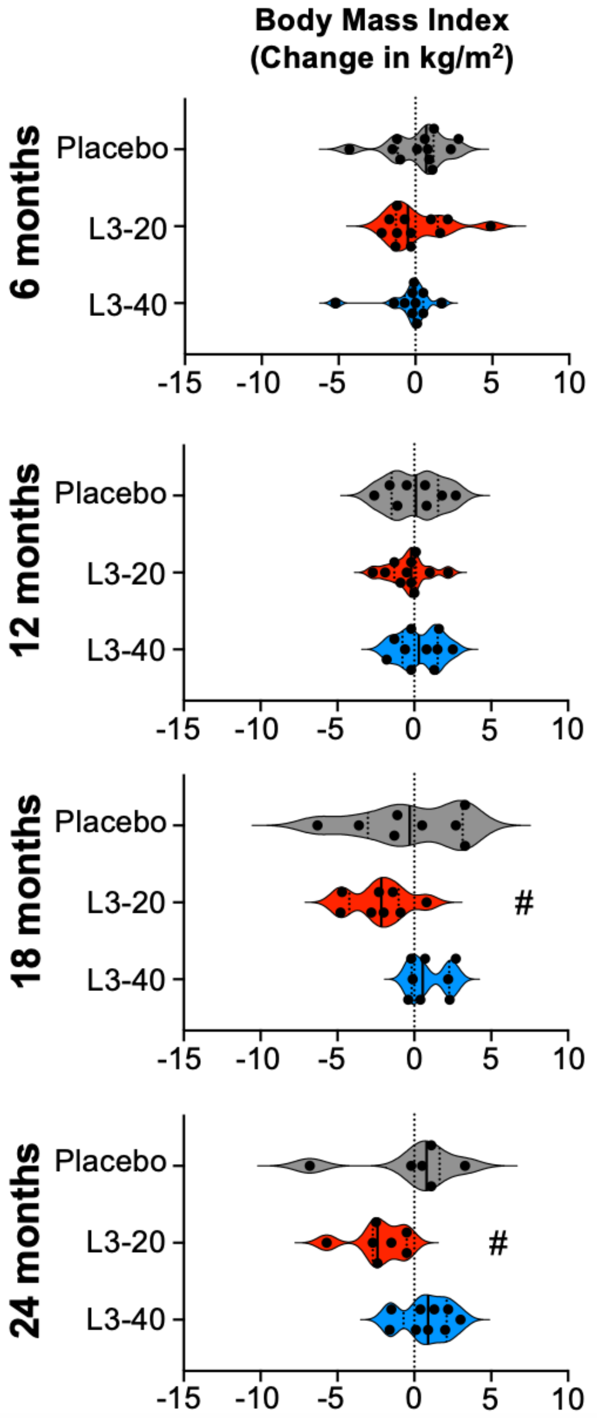

Supplementary Figure 5. Impact of experimental hookworm infection on body mass index (BMI). Changes in participant's body mass index (kg/m^2) from baseline values at each 6-monthly evaluation visit. Violin plots display each individual data point, median and IQR. Dotted vertical line indicates baseline levels (0 change). **#** Median value significant difference to baseline. Durbin, Skillings-Mack test and Wilcoxon signed-rank test: L3-20 at

18 months p=0.016, L3-20 at 24 months p=0.016. Source data are provided as a Source Data File.
