# Supplementary material for: Effect of experimental hookworm infection on insulin resistance in people at risk of type 2 diabetes
Source: Nat Commun. 2023 Jul 26;14:4503. doi: 10.1038/s41467-023-40263-4 (PMC10372076; doi:10.1038/s41467-023-40263-4)
Supplement: Supplementary file 3 — Reporting Summary [file 41467_2023_40263_MOESM3_ESM.pdf]

nature portfolio

Corresponding author(s): Dr Paul Giacomin

Last updated by author(s): Jul 4, 2023

Reporting Summary

Nature Portfolio wishes to improve the reproducibility of the work that we publish. This form provides structure for consistency and transparency in reporting. For further information on Nature Portfolio policies, see our Editorial Policies and the Editorial Policy Checklist.

Statistics

For all statistical analyses, confirm that the following items are present in the figure legend, table legend, main text, or Methods section.

n/a	Confirmed	
☐	☒	The exact sample size (n) for each experimental group/condition, given as a discrete number and unit of measurement
☐	☒	A statement on whether measurements were taken from distinct samples or whether the same sample was measured repeatedly
☐	☒	The statistical test(s) used AND whether they are one- or two-sided<br>*Only common tests should be described solely by name; describe more complex techniques in the Methods section.*
☐	☒	A description of all covariates tested
☐	☒	A description of any assumptions or corrections, such as tests of normality and adjustment for multiple comparisons
☐	☒	A full description of the statistical parameters including central tendency (e.g. means) or other basic estimates (e.g. regression coefficient) AND variation (e.g. standard deviation) or associated estimates of uncertainty (e.g. confidence intervals)
☐	☒	For null hypothesis testing, the test statistic (e.g. F, t, r) with confidence intervals, effect sizes, degrees of freedom and P value noted<br>*Give P values as exact values whenever suitable.*
☒	☐	For Bayesian analysis, information on the choice of priors and Markov chain Monte Carlo settings
☒	☐	For hierarchical and complex designs, identification of the appropriate level for tests and full reporting of outcomes
☐	☒	Estimates of effect sizes (e.g. Cohen's d, Pearson's r), indicating how they were calculated

Our web collection on statistics for biologists contains articles on many of the points above.

Software and code

Policy information about availability of computer code

Data collection	No software was used
Data analysis	GraphPad Prism 9.0 and XLSTAT for Excel (2023)

For manuscripts utilizing custom algorithms or software that are central to the research but not yet described in published literature, software must be made available to editors and reviewers. We strongly encourage code deposition in a community repository (e.g. GitHub). See the Nature Portfolio guidelines for submitting code & software for further information.

Data

Policy information about availability of data

All manuscripts must include a data availability statement. This statement should provide the following information, where applicable:

- Accession codes, unique identifiers, or web links for publicly available datasets
- A description of any restrictions on data availability
- For clinical datasets or third party data, please ensure that the statement adheres to our policy

This statement is included in the article:" The data that support the findings of this study are provided with this paper."

Human research participants

Policy information about studies involving human research participants and Sex and Gender in Research.

Reporting on sex and gender	All information about participant's sex/gender are included in the current version of the manuscript (Table 1, and methods). We have also added that both males and females participated in the abstract<br>Sex and gender was determined based on self-report by the participants.<br>Data was collected from both male and females in this study, but we did not attempt to disaggregate these results in any post-hoc analyses because the study sample size was not sufficient to draw meaningful conclusions (particularly the low numbers of males that participated)
Population characteristics	Basic demographic information about participants is included in Table 1 and Inclusion/exclusion criteria are included in the methods and study protocol:<br>"Inclusion criteria: Otherwise healthy males and females aged 18-50 years with central obesity (waist circumference> 90cm for women and >102cm for men) and increased insulin resistance as assessed via abnormal HOMA-IR>2.12 or at least two other features of metabolic syndrome determined at screening (blood pressure>135/85 mmHg, dyslipidaemia or abnormal liver function tests). Exclusion criteria, in brief, included pregnancy, established chronic disease, historical or current substance abuse, major allergies, known immunodeficiency disorder, unstable asthma or taking medications likely to interfere with study outcomes.
Recruitment	This statement in included in the manuscript:<br>"An initial recruitment target of 54 participants allowed for a dropout rate of 20%, leaving a desired recruitment target of 45 volunteers (15 in each treatment group) to participate in the trial. Volunteers who met the eligibility criteria and had given informed consent were randomly allocated on a 1:1:1 ratio according to a computer-generated sequence to one of the three study arms:"<br><br>The study protocol submitted alongside this manuscript has more specific detail about how the recruitment process was undertaken.
Ethics oversight	This statement in included in the manuscript: "The study designated C26 was approved on April 19th 2017 by the Human Research Ethics Committee of James Cook University.

Note that full information on the approval of the study protocol must also be provided in the manuscript.

Field-specific reporting

Please select the one below that is the best fit for your research. If you are not sure, read the appropriate sections before making your selection.

[x] Life sciences [ ] Behavioural & social sciences [ ] Ecological, evolutionary & environmental sciences

For a reference copy of the document with all sections, see nature.com/documents/nr-reporting-summary-flat.pdf

Life sciences study design

All studies must disclose on these points even when the disclosure is negative.

Sample size	The following information is included in the Methods section of the manuscript:<br>"In the absence of other effect size data from human trials upon which to base a power calculation, and as a change in HOMA-IR from Baseline is a primary metabolic outcome of this study, we have adapted the SUGARSPIN HOMA-IR result [29] and assume an effect size of 1.06 over 2years. A total of 15 participants in each group reflects 80% power to detect an effect size of 1.06 using the T-statistic and 1.023 using Z-statistic. The aim to recruit 54 participants allows for a potential drop-out rate of 20%."
Data exclusions	The following information is included in the current version of the manuscript: All of these exclusions were pre-established and constituted a deviation from the study protocol that compromised resultant data.<br><br>"We undertook a per-protocol analysis of metabolic outcome measures to account for the Placebo participant who received hookworm treatment in error. Data collected from the 4 participants described in the Study Progression section who, at 18 or 24 months, were retrospectively discovered to have undertaken gastric sleeve surgery or began taking metformin were excluded from the analysis. Data from one L3-20 participant whose insulin (47 mU/L), glucose (12.4 mmol/L) and HOMA-IR (26 units) values at 6 months were well beyond normal ranges and inconsistent with fasting were also excluded."
Replication	Results from this clinical trial cannot easily be replicated, without performing an additional trial

Randomization	The methods and study protocol outline the randomisation procedures:<br>"Volunteers who met the eligibility criteria and had given informed consent were block randomised (block size of 6) on a 2:2:2 ratio according to a computer-generated sequence to one of the three study arms: Placebo, 20x N. americanus L3 (L3-20) or 40x L3 (L3-40). "
Blinding	Participants and investigators were blinded to the treatment, other than the assigned producer of the inocula

Reporting for specific materials, systems and methods

We require information from authors about some types of materials, experimental systems and methods used in many studies. Here, indicate whether each material, system or method listed is relevant to your study. If you are not sure if a list item applies to your research, read the appropriate section before selecting a response.

Materials & experimental systems

n/a	Involved in the study
☒	☐ Antibodies
☒	☐ Eukaryotic cell lines
☒	☐ Palaeontology and archaeology
☒	☐ Animals and other organisms
☐	☒ Clinical data
☒	☐ Dual use research of concern

Methods

n/a	Involved in the study
☒	☐ ChIP-seq
☒	☐ Flow cytometry
☒	☐ MRI-based neuroimaging

Clinical data

Policy information about clinical studies

All manuscripts should comply with the ICMJEguidelines for publication of clinical research and a completedCONSORT checklist must be included with all submissions.

Clinical trial registration	The trial was registered with the Australian New Zealand Clinical Trials Registry (ACTRN12617000818336).
Study protocol	The study protocol has been published: doi: 10.1186/s12902-019-0461-5
Data collection	Participant confidential data, including anthropometric measures, lifestyle, and medical history, were recorded on a hardcopy Case Report Form , which was then be entered into a password protected electronic database developed specifically for this trial. Hard copy Case Report Forms were stored in participant trial folders in a locked cabinet in a secure environment in clinical trial facilities at the JCU Cairns and Townsville campuses."<br><br>Recruitment progressed from January 2018 until March 2020 and data was collected until April 2022.
Outcomes	Primary and secondary outcomes were pre-defined as per our published study protocol:<br><br>The primary outcome measure will be the safety of experimental hookworm infection of otherwise healthy women and men with 20 Na L3 as evaluated by number of reported AEs and SAEs, assessment of general health, and successful completion of the 24-month trial.<br>Secondary outcomes include:<br>1. safety/tolerability of the higher dose of 40 Na L3<br>2. longitudinal and inter-cohort changes in metabolic parameters (HOMA-IR, DEXA, WC, body mass index, fasting insulin and glucose, lipopolysaccharide-binding protein, glycated haemoglobin, blood pressure, resting heart rate, C-reactive protein, adiponectin, full blood count, white blood cell count, omega-3 poly-unsaturated fatty acid profile, urinary albumin-creatinine ratio)<br>3. immunological parameters (fluctuations in blood and serum Type 1 and Type 2 immune responses)
